# Mr. Thomas George Ouston

**Published:** 1911-08-12

**Authors:** 


MR. THOMAS GEORGE OUSTON, a prominent North Country consultant on diseases of the nose and throat, has died at Newcastle-on-Tyne, at the age of forty-two. He studied at Guy's Hospital and Leeds University, and qualified L.R.C.P. in 1891 and became Fellow of the Royal College of Surgeons in 1894. His appointments included that of surgeon to the Throat and Ear Hospital and to the Hospital for Sick Children, Newcastle-on-Tyne. He held formerly the posts of senior house surgeon and ophthalmic and aural house surgeon at the General Infirmary, Leeds, and had been a resident medical officer at the Ida Hospital, Horsforth. He was an ex-President of the Newcastle-on-Tyne Clinical Society and a member of several scientific associations.

